# Disinfection of Hands

**Published:** 1898-11

**Authors:** 


### Disinfection of Hands.

This subject, which is of such great importance in clinical surgery, has been carefully studied by Weir, whose investigations led him to the following conclusions:

1. That the solutions of corrosive sublimate are unreliable.

2. That such disinfection is far best applied, and in the order named, by the use of nascent chlorine, alcohol, or potassium permanganate.

3. That chlorine is satisfactorily evolved by the conjoined use of moistened chemical chlorinated lime and crystallized sodium carbonate.

4. That of these three procedures, the chlorine teatment is least hurtful to the hands, alcohol the most trying.

The author has devised a simple, easy and inexpensive, yet efficient method of obtaining the sterilization by nascent chlorine. After the usual scrubbing with soap and water, and the use of green soap, and cleaning the periungual spaces, one or more large crystals of carbonate of sodium (washing soda) are taken in one hand and covered with about a tablespoonful of bleaching powder (chlorinated lime), and enough water is added to make a thin paste, which at first feels warm, and from which fresh chlorine gas comes. This is rubbed for two or three minutes over the hands, nails and forearms until a creamy paste is formed, or until the sodic crystals impart a cool sensation, or until the rough grains of bleaching powder have mostly disappeared, when the hands are washed in sterile water.—*Boston Med. and Surg. Journal.*

